# Cultural Adaptation and Assessment of the Psychometric Properties of the Greek Version of the Perceived Quality of Patient-Centered Care Among Cancer Survivors

**DOI:** 10.7759/cureus.82687

**Published:** 2025-04-21

**Authors:** Nikolaos Volakakis, Theodoros Xanthos, Vasilios Raftopoulos, Magdalini Pylli, Giannoula A Kyrkou, Anna Deltsidou

**Affiliations:** 1 Oncology Unit, General Hospital of Athens "Evaggelismos", Athens, GRC; 2 Department of Midwifery, Faculty of Health and Care Sciences, University of West Attica, Athens, GRC; 3 Quality Department, Hellenic National Public Health Organization, Athens, GRC

**Keywords:** cancer care, patient centered care, patient reported experience measures, quality improvement, solid cancer

## Abstract

Background: No specific scale exists to assess the perceived quality of patient-centered care in Greek cancer survivors.

Aim: The purpose of this study was the cultural adaptation of the Quality of Patient-Centered Care (QPCC) scale and the assessment of the psychometric characteristics of the QPCC scale among Greek cancer survivors.

Sample and methods: A total of 400 cancer survivors with solid tumors were being treated at one hospital located in Athens, the capital of Greece, and participated in the validation study. The English version of QPCC was used after permission which had been obtained from the original developers. Participants completed the 48-item scale of QPCC as well as questions about sociodemographic characteristics and clinical characteristics. The validity of the scale's structure was evaluated using exploratory factor analysis, a method that identifies key dimensions measured by the tool, and the internal consistency of scale and sub-scales was estimated using Cronbach’s α.

Results: Exploratory factor analysis (Kaiser-Meyer-Olkin (KMO) = .791 and Bartlett test = 10093.336, df = 1035, p<0.01) with principal component extraction and varimax rotation revealed 10 factors with little cross-loading. Cronbach’s α for internal consistency of the whole scale was 0.946, which proved satisfactory. The lowest value of Cronbach’s coefficient was observed in the “equitable care” factor whilst the highest value was observed in the “timely care” factor. Finally, 45 items were identified. The results indicated six changes in the total of the English version such as the deletion of three items, the change of the positive wording of one item, and the transfer of two items in different factors due to better interpretation.

Conclusions: The Greek version of the QPCC scale is reliable and suitable for use among Greek patients with solid cancers.

## Introduction

The burden of cancer continues to grow globally. It is estimated that by 2040 the total number of new cancer cases will rise to 29 million due to the aging of the population [1]. Unfortunately, the long-term dramatic increase in new cases results in an uncalculated financial, social and ethical burden for patients and health systems [2].

At the beginning of 2022, the population in Greece was estimated at 10.413.982 persons [3]. The number of new cancer cases was 65,703 whilst the total number of deaths among cancer cases amounted to 32,385. Furthermore, a quarter of all deaths in Greece were attributed to cancer. The number of new cancer cases among men was 19,120 as opposed to 13,265 for women. The most frequent cancer type among women was breast cancer and among men was prostate cancer [2].

The public health sector, which serves approximately 90% of the population, is funded via compulsory social insurance. Additionally, a growing number of patients are enrolling in private insurance schemes, which are gradually expanding [4]. Recently, in 2022, a new law was passed about the reengineering of primary health care services. The new gatekeeping system includes specific terms and conditions for registering with general practitioners. All the inhabitants should be assigned to a general health practitioner of their choice, who guides them in the health care system as well as to fill in their electronic medical record [5].

The most common barriers faced by Greek cancer survivors include: a) the lack of a holistic health approach, to cover the spectrum of the disease; b) fragmented services; b) the lack of precise data to capture the true size of the problem; since there is no epidemiological surveillance system in use; c) limited palliative health care services; and d) the absence of a linkage between secondary and primary health care [6].

The provision of high-quality care is a challenge for all the stakeholders. Patient-centeredness of care is one of the pillars of the quality of health care [7]. Τhe perceived quality of patient-centered care (QPCC) is defined as the degree to which healthcare services fulfill the principles of patient-centered care (PCC) [8]. In particular, the six dimensions of PCC are described as) respecting patients' values, preferences, and needs; b) providing coordinated and comprehensive care; c) providing information and education; d) supporting physical comfort; e) providing emotional support and alleviating feelings of fear and anxiety; and f) encouraging family and significant others participation in clinical decision making [7].

The perceived QPCC is assessed by using a specific patient-reported experience measure. According to the literature, only one scale exists, which assesses the quality of perceived patient-centered care [9,10]. Initially, a 48-item scale had been developed and validated among hematologic cancer survivors in Australia [10]. The Australian version of the QPCC scale consists of 10 factors and 48 items; 46 items load on 10 factors and two items are independent as they do not load any factor. The factors include timely care, respectful communication, cancer information, treatment decision-making, treatment delivery, respect for patient preferences and values, equitable care, coordinated and integrated care, emotional support, and follow-up care [11]. Later, the scale was validated among Spanish cancer survivors with solid cancers [12]. The Spanish version of QPCC consisted of 30 items and five factors; timely care, clarity of information for treatment decision-making, information for treatment decision-making, activities to address biopsychological needs, and respectful and coordinated care [12]. 

To assess how Greek cancer survivors perceive the cancer care they receive in public hospitals, a reliable and valid tool is needed. To our knowledge, there is no reliable tool to evaluate the perceived QPCC among Greek cancer survivors. The purpose of this study was twofold: the culture adaptation of the QPCC scale and the assessment of its psychometric characteristics.

## Materials and methods

Study design

This is a validation study of the perceived QPCC scale in Greek cancer survivors. Data collection took place from September 2023 to March 2023. Patient clinical data were retrieved from medical records after authorized access by the researcher. Cancer survivors visiting the outpatient clinic were screened if they met the eligibility criteria. The researcher asked eligible participants to complete a questionnaire while receiving their treatment. Also, patients were able to fill out the questionnaire at home and return it during their next appointment.

Measurement tool

The English version of QPCC was used [11]. Permission to use this scale was obtained from the original developer. Two bilingual experts with experience in medical terminology conducted the translation and back translation of the initial Australian version of the QPCC scale [13,14]. Finally, a panel of five experts evaluated the translations and assessed face and content validity. According to the literature, to ensure content validity five experts are a satisfactory number [15]. The team of experts consisted of a researcher, two academics with experience in the validation of psychometric scales, and two internists-oncologists. A pilot study using five cancer survivors followed to make proper changes in the wording or syntax.

The first part of the questionnaire included the 48-item QPCC scale, as the original scale. The rating for each item, ranged from 1 to 5 (strongly agree, agree, disagree, strongly disagree, not applicable to me). The second part of the questionnaire included sociodemographic information such as age, gender, education level, marital status, place of residence, current occupation, and living conditions. The third part included questions about the clinical status such as time since diagnosis, Eastern Cooperative Oncology Group Performance Status Scale (ECOG PS scale), clinical stage of the disease, type of cancer, past therapies, and current therapies.

Sample

The sample was a convenient consecutive sample of cancer survivors with solid cancers who received medical treatment in a one-day clinic of a large public hospital in Athens. Participants were eligible if they had been diagnosed with solid cancer and undergoing anticancer therapies, were willing to participate, spoke and understood the Greek language, and were in good psychological or physical condition. Patients who had an ECOG PS score of 4 were excluded from the recruitment. The sample size was 400 patients, selected from a single public hospital in Athens, the capital of Greece.

Ethical considerations

Ethical approval was obtained by the Research Ethics Committee of the University of West Attica (Reference Number 84630, September 22, 2023). Additionally, the study received approval from the Scientific Council of the hospital (Reference Number 13392, July 19, 2023). The researcher informed all the participants about the aim of the study, and that they had to sign an informed consent form. The participants were then asked to complete the questionnaire.

Statistical analysis

All items were coded and scored by the researcher, and the questionnaires were included in the data set. SPSS for Windows, version 29.0.2.0 (IBM Corp., Armonk, NY, USA) was used for the analysis. The frequencies, mean values, and medians for sociodemographic and clinical characteristics were calculated. Also, the frequencies and the percentages of all items were counted. Exploratory factor analysis with principal component extraction and varimax rotation was performed. The number of factors was verified using the eigenvalue criterion, and the scree plot was assessed. Four hundred participants are considered an adequate number for factor analysis [16]. However, the Kaiser-Meyer-Olkin test and the Barlett test of sphericity were used to assess the adequacy of the sample for the current factor analysis. The factor’s loading was estimated. A factor loading between 0.3 to 0.4 is minimally acceptable [17]. However, a lower cut-off value of more than 0.3 can be accepted if it is deemed very important for the interpretation of the factor.

## Results

Sample size

A total of 400 patients responded to the 48 questions on this scale, resulting in a ratio of eight participants per question, which is considered satisfactory for factor analysis [18]. The appropriateness of the sample was further validated by the Kaiser-Meyer-Olkin (KMO) index, which was calculated at .791. Additionally, Bartlett's test of sphericity yielded a value of 10093.336 (df = 1035), confirming that the data is suitable for factor analysis.

Socio-demographic characteristics

All the eligible participants agreed to fill in the questionnaire. The mean age of the patients was 65.86 (sd=11.54). One in two participants (56%) were men. Most of the patients originated from Greece, had completed secondary education, and lived in Athens (Table 1).

**Table 1:** Frequences and percentages of sociodemographic characteristics (Ν=400)

	Ν	%
Gender		
Men	227	56.8
Women	173	43.2
Nationality		
Greek	367	91.8
Non-Greek	3	7.8
N/A	2	0.4
Education level		
Some Primary School Classes	123	30.8
Primary School	67	16.8
Secondary School	82	20.5
High School	51	12.8
Post-Secondary	65	16.2
University	8	2
Post University studies	2	0.5
N/A	2	0.5
Marital status		
Married	226	56.5
Unmarried	62	15.5
Widower	18	4.5
Cohabitation	82	20.5
Divorced	3	0.8
Ν/Α	3	2.2
Occupation		
Public civil servant	81	20.3
Private employee	82	20.5
Unemployment	79	19.7
Freelance	66	16.5
Student	10	2.5
Retired	55	13.7
N/A	27	6.8
Place of residence		
Urban	305	76.3
Rural	90	22.5
N/A	5	1.2
Living condition		
Alone	84	21.0
With my partner	87	21.8
With my family	224	56
Waith a carer	1	0.2
N/A	4	1.0

Clinical characteristics

One out of two patients had a gastrointestinal malignancy followed by patients with gynecological cancer. Most of the patients (70%) had an ECOG PS score of 1. In terms of types of therapy, 75% of the participants had received hormone therapy or a combination of immunotherapy and chemotherapy in the past while 68, 5% were receiving chemotherapy during the period of data collection. The median time since diagnosis was 12 months (IQR 4 - 25 months) (Table 2).

**Table 2:** Frequencies and percentages of clinical characteristics (Ν=400)

	Ν	%
ECOGSTATUS		
Level 0	46	11.5
Level 1	292	73.0
Level 2	59	14.8
Level 3	3	0.7
Type of cancer		
Ca Gynecologic	78	19.5
Ca Gastrointestinal	209	52.3
Ca Urinary system	57	14.2
Other type	56	19.5
Stage of disease		
Stage 0	14	3.5
Stage 1	106	26.5
Stage 2	148	37.0
Stage 3	81	20.3
Stage 4	51	12.7
Past therapies		
No cure	48	12
Lumpectomy surgery	6	1.5
Hormone therapy	150	37.5
Chemotherapy	4	1.0
Immunotherapy	8	2.0
Targeted therapy	4	1.0
Radiotherapy/Chemotherapy	34	8.5
Immunotherapy/Chemotherapy	146	36.5
Current therapy		
No cure	20	5.0
Lumpectomy surgery	2	0.5
Hormone therapy	32	8.0
Immunotherapy	46	11.5
Chemotherapy	274	68.5
Targeted therapy	2	0.5
Radiotherapy/Chemotherapy	6	1.5
Immunotherapy/Chemotherapy	18	4.5

Reliability analysis

Cronbach’s alpha values for each subscale ranged from 0.52 to 0.93, while the total Cronbach’s alpha was 0.95. The "timely care" subscale exhibited a high Cronbach’s alpha of .93, indicating excellent internal consistency. Conversely, Cronbach’s alpha for "equitable care" was .52, suggesting the need for rewording one item to better reflect the content of this factor (Table 3).

**Table 3:** Internal consistency of the subscales and the total scale

Factors	Cronbach’s alpha
Coordinated and integrated care	0.82
Cancer information	0.79
Timely care	0.93
Treatment decision making	0.82
Follow up care	0.75
Treatment delivery	0.82
Patient preferences and values	0.73
Respectful communication	0.67
Emotional support	0.67
Equitable care	0.52
Total scale	0.95

Factor analysis

A total of 10 factors were identified, accounting for 69.94% of the total variation, which is considered a very satisfactory percentage. Although questions 11, 32, 33, 40, and 46 had factor loading values lower than 0.3, these items were retained because the model fit, and reliability remained satisfactory (Table 4) [18].

**Table 4:** Factor analysis of Quality of Patient-Centered Care (QPCC) scale

	Factor loading	Communality	% of the total variance
TIMELY CARE (factor 3)			13.817
1.I had to wait too long from Getting a referral to a cancer doctor to my first visit with him/her	.919	.873	
2. I had to wait too long from getting a referral to a cancer doctor to my first visit with him/her	.935	.900	
3. I had to wait too long from my first visit with the cancer doctor to get my cancer diagnosis	.920	.884	
4. I had to wait too long from getting my cancer diagnosis to starting my first cancer treatment (e.g. chemotherapy)	.817	.848	
RESPECTFUL COMMUNICATION (factor 8)			5.448
The staff at the hospital			
5. Showed respect for me	.677	.221	
6.The staff at the hospital Showed respect for my family or friends	.442	.247	
7.The staff at the hospital Talked to me in a way I could understand	.540	.290	
CLEAR INFORMATION ABOUT THE DISEASE (factor 2)			6.812
The staff at the hospital gave me			
8. Information about cancer that was easy to understand	.852	.448	
9.A list of questions that cancer patients commonly ask	.757	.422	
10. Information about cancer and treatments to take home (e.g. booklets. websites)	.573	.248	
TREATMENT DECISION MAKING (factor 4)			11.065
The doctors at the hospital explained to me			
11. All the treatments I could have	.212	.352	
12. The consequences of not having treatment	.391	.390	
13.The short-term side effects of each treatment option	.332	.549	
14.The long-term side effects of each treatment option	.663	.491	
15. How each treatment option might affect my length of life	.730	.491	
16.I could get a second medical opinion if I wanted to	.663	.464	
17. When I was making my most recent treatment decision, doctors at the hospital, gave me the time I needed to consider all my treatment options before making a decision	.601	.651	
18. When I was making my most recent treatment decision, doctors at the hospital, involved my family or friends in decision making about my care when I wanted them to	.597	.569	
TREATMENT DELIVERY (factor 6)			4.415
During my treatment, the staff of the hospital			
19. made sure I received the treatment I was meant to have	.248	.250	
20.Make sure I don’t receive unnecessary treatment or diagnostic tests	.349	.584	
21. Chose the evidence-based treatment for my case	.642	.363	
22. Responded promptly to my pain or discomfort	.283	.447	
23. Had up to date information about my cancer care	.553	.460	
24. Had up to date information about my cancer care	.761	.364	
25. Had up to date information about my cancer care	.738	.454	
RESPECT FOR PATIENT’S VALUES. PREFERENCES (factor 7)			7.108
During my treatment I was able to choose which			
26. Hospital provided my treatment	.686	.525	
27. Hospital provided my treatment	.753	.588	
28. Doctor, I saw each appointment	.718	.819	
EQUITABLE CARE (factor 10)			5.393
The treatment I received at the hospital			
29. was too expensive for me	.765	.832	
30. Too far away from where I lived	.579	.863	
EMMOTIONAL SUPPORT (factor 9)			4.809
The staff at the hospital helped me			
31. Deal with being worried, upset, or sad	.261	.423	
32. Deal with my spiritual needs	.109	.874	
33. Deal with changes in my personal relationships	.084	.783	
40. Deal with being worried, upset or sad	-.054	.817	
INTERGRATED CARE (factor 1)			6.122
The staff at the hospital helped me			
34. Deal with day-to-day tasks (e.g. childcare, housework)	.479	.567	
35. Get financial assistance	.533	.801	
36. Organize transport to and from the hospital	.763	.772	
37. Get accommodation close to the hospital	.775	.858	
38. Get parking at the hospital that was affordable	.751	.850	
39. Find other cancer patients I could talk to about their cancer experiences	.630	.803	
41. Find others in a similar situation to talk to	.461	.827	
FOLLOW UP CARE (factor 5)			4.952
42. After treatment had ended, staff at the hospital explained to me: What to expect during follow-up tests	.775	.459	
43. Who to contact if I have questions about my care	.679	.418	
44. When should I seek medical advice (e.g. if I had an unexpected side-effect)	.664	.424	
45. What I could do to be well	.716	.454	
46. How to manage my care at home	.235	.368	

Six changes were made to the original scale to better align it with the cultural and contextual framework of the Greek healthcare system. For example, the item "I waited a long time for my first visit" was removed from timely care due to its limited relevance in the current healthcare system. The removal of this item did not significantly impact Cronbach's alpha, which slightly decreased from 0.93 to 0.91. Additionally, item 38, "the staff at the hospital helped me/my partner find affordable parking", was excluded from the subscale of coordinated and integrated care.

The item “the staff at the hospital helped my family find others in a similar situation” was moved from the factor “coordinated and integrated care” to the factor “emotional support”. This adjustment not only improved the Cronbach’s alpha, but also better interpreted the factor “emotional support”. Also, by removing this item from “coordinated and integrated care”, no significant change in Cronbach’s alpha was observed. In place of this item we moved the question “after treatment had ended, staff at the hospital helped me move smoothly between different hospitals, clinics, or health services”, which in the Australian version didn’t load any factor.

The positive wording for question 29, "the treatment I received at the hospital was too expensive for me”, as "the treatment I received at the hospital was not too expensive for me" was revised because half of the participants responded “not applicable”, due to misunderstanding. This modification also resulted in an increase in the Cronbach's alpha for the equitable care subscale.

Lastly, item 47, “after treatment had ended, staff at the hospital helped me move smoothly back home”, which in the Australian version didn’t load any factor, was removed from the Greek scale.

The loading values of the questions per factor, the communality of variance of each question and the percentage of variance of the factors are listed in Table 4.

## Discussion

To our knowledge, this is the first study that the QPCC scale validated in Greek setting. It is a reliable tool and has very good psychometric properties and it could be used to assess the perceived quality of patient centered cancer care in the Greek language.

According to our analysis the Greek version of the scale consists of 10 factors and 45 items. In parallel, the English version consisted of 10 factors and 48 items whilst the Spanish version has five factors and 30 items [11,12].

The internal consistency of perceived QPCC scale ranged from .52 to .93 for each subscale whilst the Cronbach’s alpha of the total scale was .93. Our values of subscales are lower than the values of Australian version (Cronbach's alpha = .73 to .94) as well as the Spanish version (Cronbach's alpha = .73 to .90). More precisely, in our analysis the lowest value of Cronbach’s alpha was observed in "equitable care" (.52) and the highest value in “timely care” (.93), whilst the lowest and highest value of Australian version were observed in “coordinated and integrated care” and “timely care” respectively. On the other side, the lowest and highest value of the Spanish version were observed in “activities to address biopsychosocial needs” (.90) and in “respectful and coordinated care” (.73) respectively [11,12]. However, the values of our study are considered satisfactory [19]. Additionally, Cronbach’s alpha of the total scale of the Greek version is .93 and thus similar to the two other versions, .94 for the Australian version and .90 for the Spanish version [11,12].

According to our analysis, the factors “clear information about the disease”, “respect to the values”, “references & expressed needs of patients”, “respectful communication”, “follow up care”, “treatment delivery”, “information for treatment decision making”, proposed to remain as they stand in the English version of the questionnaire. Minor changes have been made in the factors “timely care”, “coordinated and integrated care”, “emotional support” and “equitable care”.

The specific characteristics of the Greek healthcare system, such as the absence of a comprehensive referral process and free access to cancer treatment, likely influenced responses to items related to equitable and timely care. The socio-demographic characteristics of the study population played a key role in the final modification of the scale. Most of the participants were men, married, and living with their family, thus they had a supportive environment and suffered from gastrointestinal cancer. In contrast, among Spanish participants, the majority were women with breast cancer [12] whilst Australian participants suffered from haematological malignancies [11]. The Spanish and Greek participants shared similar educational levels, compared to Australian participants where the majority had completed a high education degree [20].

The decision to modify the positive wording in item "the treatment I received at the hospital was too expensive for me" was due to a misunderstanding. The cancer treatment in Greece is fully covered by the National Agency for the Provision of Health Services for insured individuals and by the special budget of the Ministry of Health for uninsured individuals. So, patients do not directly experience the financial effects of their treatment.

The item, “I waited a long time for my first visit to my doctor/my personal doctor" was excluded from “timely care” as it does not seem to be applicable in the Greek health care context. The system of referrals from primary health care services is in its infancy. The relevant law was passed in 2023, and an effective referral system has not yet been set up. Individuals can book any appointment directly with the specialist of their choice in the public or private sector.

It is needless to say that patients do not leave the hospital unless they feel well. The health care professionals are proceeding with all the necessary actions for the recovery of patients. There is not in place a specific procedure to ensure the smooth transition to home from the hospital for cancer survivors who are undergoing anti-cancer treatments in outpatient clinics. So, we suggest the deletion of the item “after the end of my treatment, the staff of the hospital helped to move smoothly back home” due to misunderstanding.

Additionally, the item "after the end of my treatment, the hospital staff facilitated the smooth connection with different clinics, departments, services", which in the Australian version didn’t load any factor, was moved to “coordinated care” because of its high importance in the Greek healthcare provision framework [11].

Finally, the deletion of item 38, “the staff at the hospital helped me get parking at the hospital that was affordable”, was deemed necessary. Few hospitals offer parking near the hospitals, and this is not of high importance for the patients in their rating regarding the quality of health care. This finding is in line with other researchers [21,22].

In summary, the Greek version of perceived QPCC is a 45-item tool that consists of 10 factors. More specifically, timely care contains three items (Q1-Q3), respectful communication three items (Q4-Q6), cancer information three items (Q7-Q9), treatment decision making eight items (Q10- Q17), treatment delivery seven items (Q18-Q24), emotional support five items (Q25-Q29), respect to the patient values and expressed needs three items (Q30-Q32), equitable care two items (Q33, Q34), coordinated and integrated care six items (Q35-Q40) and at least the follow up care includes five items (Q41-Q45) (Appendix).

Strengths and limitations

To our knowledge, this is the first validation study of the QPCC scale among Greek cancer survivors with solid malignancies. The study was conducted in a single hospital in Athens, which may not accurately represent the experiences of patients in rural areas or private healthcare settings. Nonetheless, in Greece, anticancer care is predominantly provided by large public hospitals in key urban locations. The hospital and thus the clinic where this research was conducted is among the largest in Athens and provides services to patients with various types of solid tumors from Athens and surrounding cities and regions. Additionally, the study included patients who had received health services in the past and those who may have received outpatient care in other settings. A confirmatory factor analysis as well as a test-retest reliability assessment of the QPCC were not our aims given that it is our future intention to assess them in a sample of women suffering from breast cancer.

## Conclusions

Globally, patient-centered care remains a significant challenge for healthcare systems. The QPCC scale demonstrates acceptable Cronbach’s alpha values and shows acceptable validity and reliability. It is considered as an easy-for-use and reliable instrument for assessing the perceived QPCC in Greek cancer survivors. Further studies to assess the perceived patient-centeredness of healthcare services are essential, as emphasized by international organizations. The evaluation of the perceived quality of cancer care in both public and private hospitals, covering both urban and rural areas, is an imperative need. The assessment of the perceived QPCC will guide evidence-based interventions aimed at quality improvement of cancer care services.

## Appendices

**Table 5:** SCALE OF ASSESMENT OF PERCEIVED QUALITY OF PATIENT CENTERED CARE IN GREEK LANGUAGE

Παρακάτω κυκλώστε την απάντηση σε κάθε ερώτηση που περιγράφει καλύτερα την εμπειρία σας/ Below, circle the answer to each question that best describes your experience. Οι ερωτήσεις που ακολουθούν αναφέρονται στις υπηρεσίες που δεχθήκατε από την πρώτη στιγμή που είδατε κάποιον ιατρό μέχρι την έναρξη της αντινεοπλασματικής θεραπείας/ The next questions ask about the cancer care you received from the time you first saw your general practitioner about cancer-related symptoms or had cancer screening until the start of your cancer treatment.					
Παράγοντες/Ερωτήσεις/Factors/items	Συμφωνώ απόλυτα/Strongly agree Ν (%)	Συμφωνώ/Agree Ν (%)	Διαφωνώ/ Disagree Ν (%)	Διαφωνώ απόλυτα/ Strongly disagree Ν (%)	Δεν ισχύει στην περίπτωση μου/ Not applicable to me Ν (%)
A.ΕΓΚΑΙΡΗ ΦΡΟΝΤΙΔΑ/ΤΙΜΕLY CARE					
Περίμενα μεγάλο χρονικό διάστημα/ I had to wait too long:					
1. Για να κλείσω ραντεβού με τον ογκολόγο ιατρό/ to get a referral to a cancer doctor	1	2	3	4	5
2. Για να τεθεί η οριστική διάγνωση/ for my first visit with the cancer doctor to get my cancer diagnosis	1	2	3	4	5
3. Για να ξεκινήσω την αντινεοπλασματική θεραπεία/ between getting my cancer diagnosis and starting my first cancer treatment (e.g. chemotherapy)	1	2	3	4	5
Οι επόμενες ερωτήσεις αφορούν στη συνολική φροντίδα που έχετε λάβει από το νοσοκομείο/ The next questions ask about the overall cancer care you received at the hospital where you received most of your treatment					
Β. ΕΠΙΚΟΙΝΩΝΙΑ ΜΕ ΣΕΒΑΣΜΟ/ RESPECTFUL COMMUNICATION					
Το προσωπικό του νοσοκομείου/ The staff at the hospital:					
4. Με σέβεται/ showed respect to me	1	2	3	4	5
5. Σέβεται τον/την συνοδό μου/ showed respect for my family or friends	1	2	3	4	5
6. Μου μίλησε με απλό και κατανοητό τρόπο/ talked to me in a way I could understand	1	2	3	4	5
Γ. ΕΝΗΜΕΡΩΣΗ ΓΙΑ ΤΟ ΝΟΣΗΜΑ/ CANCER INFORMATION					
Το προσωπικό του νοσοκομείου/ The staff at the hospital:					
7. Με ενημέρωσε με κατανοητό τρόπο σχετικά με το νόσημά μου/ Informed me about cancer in a way that was easy to understand	1	2	3	4	5
8. Με ρώτησε αν έχω κάποιες απορίες σχετικές με το νόσημά μου/ gave me a list of questions that cancer patients commonly ask	1	2	3	4	5
9. Με ενημέρωσε σχετικά με το που θα μπορούσα να βρω επιπρόσθετες πληροφορίες για το νόσημα μου και τις θεραπείες μου/ gave me information about cancer and treatments to take home (e.g. booklets, websites)	1	2	3	4	5
Δ.ΕΝΗΜΕΡΩΣΗ ΓΙΑ ΤΗ ΛΗΨΗ ΑΠΟΦΑΣΗΣ ΣΧΕΤΙΚΑ ΜΕ ΤΗ ΘΕΡΑΠΕΙΑ/ TREATMENT DECISION MAKING					
Οι γιατροί με ενημέρωσαν αναλυτικά/ The doctors at the hospital explained to me:					
10. Για όλες τις διαθέσιμες θεραπείες που θα μπορούσα να έχω/ all of the treatments I could have	1	2	3	4	5
11. Την εξέλιξη της νόσου χωρίς θεραπεία/ the consequences of not having treatment	1	2	3	4	5
12. Τις άμεσες παρενέργειες από τη θεραπεία/ the short-term side effects of each treatment option	1	2	3	4	5
13. Τις μακροχρόνιες παρενέργειες από τη θεραπεία/ the long-term side effects of each treatment option	1	2	3	4	5
14. Πως θα μπορούσε η κάθε θεραπεία θεραπευτικό σχήμα να επηρεάσει το προσδόκιμο της ζωής μου/ How each treatment option might affect my length of life	1	2	3	4	5
15. Ότι θα είμαι ελεύθερος/η να ζητήσω και μια δεύτερη ιατρική γνώμη/ I could get a second medical opinion if I wanted to	1	2	3	4	5
16. Μου έδωσαν το χρόνο που χρειαζόμουν για να αξιολογήσω όλες τις θεραπευτικές επιλογές που είχα, πριν λάβω την οριστική απόφαση για τη θεραπεία μου/ Gave me the time I needed to consider all my treatment options before making a decision	1	2	3	4	5
17. Ενημέρωσαν τον/την συνοδό μου, ότι θα μπορούσε να συμμετέχει στη λήψη απόφασης σχετικά με τη θεραπεία μου, αν το επιθυμούσα/ Involved my family or friends in decision making about my care when I wanted them to	1	2	3	4	5
Ε. ΠΑΡΟΧΗ ΘΕΡΑΠΕΙΑΣ/ TREATMENT DELIVERY					
Κατά τη διάρκεια της θεραπείας μου, το προσωπικό του νοσοκομείου:/ During treatment, staff at the hospital:					
18. Φρόντισε να λάβω τη θεραπεία που είχε συνταγογραφηθεί/ made sure I received the treatment I was meant to have	1	2	3	4	5
19. Φρόντισε να μην κάνω θεραπείες ή εξετάσεις που είναι περιττές/ made sure I don’t receive unnecessary treatment or diagnostic tests	1	2	3	4	5
20. Επέλεξε την επιστημονικά τεκμηριωμένη θεραπεία για την περίπτωση μου/ made sure I received evidence-based treatment	1	2	3	4	5
21. Ανταποκρίνονταν κατάλληλα όταν πονούσα ή δεν ένιωθα καλά/ attended promptly to my pain or discomfort	1	2	3	4	5
22. Ήταν επαρκώς ενημερωμένο για την περίπτωση μου/ had up-to-date information about my cancer care	1	2	3	4	5
23. Μου απαντούσε με συνέπεια για τα ζητήματα σχετικά με τη θεραπεία μου, χωρίς να με μπερδεύει/ gave me consistent information about my treatment	1	2	3	4	5
24. Έκλεινε τα ραντεβού μου με τέτοιο τρόπο, ώστε να μη χρειάζεται να επισκέπτομαι το νοσοκομείο περισσότερες φορές από το αναγκαίο/ coordinated my appointments so that I did not have to go to hospital more than necessary	1	2	3	4	5
ΣΤ. ΣΥΝΑΙΣΘΗΜΑΤΙΚΗ ΣΤΗΡΙΞΗ/ EMOTIONAL SUPPORT					
Το προσωπικό του νοσοκομείου βοήθησε/ The staff at the hospital helped:					
25. Να διαχειριστώ τις ανησυχίες ή το άγχος μου ή τις αρνητικές μου σκέψεις/ deal with being worried, upset, or sad	1	2	3	4	5
26. Να υποστηρίξω τις θρησκευτικές μου ανάγκες/ deal with my spiritual needs	1	2	3	4	5
27. Να αντιμετωπίσω τις αλλαγές στις προσωπικές μου σχέσεις/ deal with changes in my personal relationships	1	2	3	4	5
28. Τον/ την συνοδό μου να διαχειριστεί το άγχος ή τις ανησυχίες ή τις αρνητικές του σκέψεις/ family or friends deal with being worried, upset, or sad	1	2	3	4	5
29. Τον/την συνοδό μου να συζητήσει με τους συνοδούς άλλων ασθενών/ family or friends to find others in a similar situation to talk to	1	2	3	4	5
Ζ.ΣΕΒΑΣΜΟΣ ΣΤΙΣ ΑΞΙΕΣ, ΠΡΟΤΙΜΗΣΕΙΣ ΚΑΙ ΕΚΦΡΑΣΜΕΝΕΣ ΑΝΑΓΚΕΣ ΤΩΝ ΑΣΘΕΝΩΝ/ PATIENT PREFERENCES AND VALUES					
Κατά τη διάρκεια της θεραπείας μου, είχα τη δυνατότητα να επιλέξω/ During my treatment, I was able to choose which:					
30. Το νοσοκομείο που θα λάβω τη θεραπεία μου/ hospital provided my treatment	1	2	3	4	5
31. Το γιατρό που θα με παρακολουθεί/ doctor provided my treatment	1	2	3	4	5
32. Το γιατρό που θα αντιμετωπίζει τις παρενέργειες από τη θεραπεία μου/ doctor I saw for each appointment	1	2	3	4	5
Η. ΙΣΟΤΙΜΗ ΦΡΟΝΤΙΔΑ/ EQUITABLE CARE					
33. Η θεραπεία ΔΕΝ με επιβάρυνε οικονομικά/ The treatment Ι received at the hospital was not too expensive	1	2	3	4	5
34. Το νοσοκομείο, όπου έλαβα τη θεραπεία μου, ήταν μακριά από τον τόπο διαμονής μου/ The hospital where I received my treatment was too far away from where I lived	1	2	3	4	5
Η.ΟΛΟΚΛΗΡΩΜΕΝΗ ΚΑΙ ΣΥΝΤΟΝΙΣΜΕΝΗ ΦΡΟΝΤΙΔΑ/ COORDINATED AND INTERGRATED CARE					
Το προσωπικό του νοσοκομείου βοήθησε/ The staff at the hospital helped me:					
35. Να διατηρήσω τις καθημερινές μου δραστηριότητες/ deal with day-to-day tasks	1	2	3	4	5
36. Να κάνω τις απαραίτητες ενέργειες για να λάβω τις παροχές που δικαιούμαι (οικονομικά βοηθήματα, φοροελαφρύνσεις, άλλες παροχές)/ get financial assistance	1	2	3	4	5
37. Δίνοντάς μου πληροφορίες για την μετακίνησή μου από και προς το νοσοκομείο/ giving me information about my transportation to and from the hospital	1	2	3	4	5
38. Δίνοντάς μου πληροφορίες για τη δυνατότητα διαμονής κοντά στο νοσοκομείο/ get accommodation close to the hospital	1	2	3	4	5
39. Μετά το τέλος της θεραπείας, διευκόλυνε τη διασύνδεση μου σε διαφορετικές κλινικές ή νοσοκομεία ή άλλες υπηρεσίες (πχ κοινωνικές υπηρεσίες)/ after the termination of the treatment, to move smoothly between different hospitals, clinics or health services	1	2	3	4	5
40. Να έρθω σε επαφή με άλλους ασθενείς ή συλλόγους ασθενών/ find other cancer patients I could talk to about their cancer experiences	1	2	3	4	5
Θ. ΣΥΝΕΧΙΖΟΜΕΝΗ ΦΡΟΝΤΙΔΑ/ FOLLOW-UP CARE					
Μετά το τέλος της θεραπείας, το προσωπικό του νοσοκομείου με ενημέρωσε/ After the treatment had ended, staff at the hospital explained to me:					
41. Για τα επόμενα βήματα (επαναληπτικές εξετάσεις)/ what to expect during follow-up tests	1	2	3	4	5
42. Με ποιον θα μπορούσα να επικοινωνήσω για οποιαδήποτε απορία είχα σχετικά με τη φροντίδα μου/ who to contact if I had questions about my care	1	2	3	4	5
43. Για την βαρύτητα των ανεπιθύμητων ενεργειών και σε ποιες περιπτώσεις θα έπρεπε να ζητήσω άμεσα ιατρική συμβουλή/ when I should seek medical advice (e.g. if I had an unexpected side-effect)	1	2	3	4	5
44. Τι θα έπρεπε να κάνω για να βελτιώσω την υγεία μου/ what I could do to be well	1	2	3	4	5
45. Ενημέρωσε το/τη συνοδό μου για όλα τα θέματα σχετικά με την συνέχιση της θεραπείας μου στο σπίτι/ how to manage my care at home	1	2	3	4	5

## References

[1] (2024). The Burden of Cancer. https://canceratlas.cancer.org/the-burden/the-burden-of-cancer.

[2] (2024). GLOBOCAN 2022 All Cancers Fact Sheet. https://gco.iarc.who.int/media/globocan/factsheets/cancers/39-all-cancers-fact-sheet.pdf.

[3] (2024). Data on Estimated Population (1.1.2023) and Migration Flows (2022). https://www.statistics.gr/documents/20181/de3e26f6-9b77-d2e5-2ca3-e13bcafe482a.

[4] (2024). Greece: Country Health Profile 2023. https://www.oecd.org/en/publications/greece-country-health-profile-2023_dd530c3e-en.html.

[5] (2024). Law 4931_2022_Doctor for all, equal and quality access to the services of the National Organization for the Provision of Health Services and Primary Health Care and other urgent provisions. https://www.moh.gov.gr/articles/health/dieythynsh-prwtobathmias-frontidas-ygeias/nomothesia-prwtobathmias-frontidas-ygeias/10511-n-4931-2022-giatros-gia-oloys-isotimh-kai-poiotikh-prosbash-stis-yphresies-toy-ethnikoy-organismoy-paroxhs-yphresiwn-ygeias-kai-sthn-prwtobathmia-frontida-ygeias-kai-alles-epeigoyses-diatakseis.

[6] (2024). Country Cancer Profile. Greece. https://www.oecd.org/content/dam/oecd/en/publications/reports/2023/02/eu-country-cancer-profile-greece-2023_e4e73f2c/30b7e1f9-en.pdf.

[7] Institute of Medicine (US) Committee on Quality of Health Care in America (2001). Crossing the Quality Chasm. A New Health System for the 21st Century.

[8] (2020). 2019 National Healthcare Quality and Disparities Report. https://www.ncbi.nlm.nih.gov/books/NBK579354/.

[9] Tzelepis F, Rose SK, Sanson-Fisher RW, Clinton-McHarg T, Carey ML, Paul CL (2014). Are we missing the Institute of Medicine's mark? A systematic review of patient-reported outcome measures assessing quality of patient-centred cancer care. BMC Cancer.

[10] Volakakis N, Pylli M, Raftopoulos V, Kyrkou I, Xanthos T, Deltsidou A (2024). Exploration of the factors that influence perceived quality of patient centered care among cancer survivors: a systematic review. Eur J Oncol Nurs.

[11] Tzelepis F, Sanson-Fisher RW, Hall AE, Carey ML, Paul CL, Clinton-McHarg T (2015). Development and psychometric evaluation of the Quality of Patient-Centered Cancer Care measure with hematological cancer survivors. Cancer.

[12] Doubova SV, Martinez-Vega IP, Gutiérrez-De-la-Barrera M (2020). Psychometric validation of a Patient-Centred Quality of Cancer Care questionnaire in Mexico. BMJ Open.

[13] Chen HY, Boore JR (2010). Translation and back-translation in qualitative nursing research: methodological review. J Clin Nurs.

[14] Sousa VD, Rojjanasrirat W (2011). Translation, adaptation and validation of instruments or scales for use in cross-cultural health care research: a clear and user-friendly guideline. J Eval Clin Pract.

[15] Lynn MR (1986). Determination and quantification of content validity. Nurs Res.

[16] Comrey AL, Lee HB (1992). A First Course in Factor Analysis. Second edition. https://doi.org/10.4324/9781315827506.

[17] Yusoff MS, Arifin WN, Hadie SN (2021). ABC of questionnaire development and validation for survey research. Educ Med J.

[18] Streiner DL (1994). Figuring out factors: the use and misuse of factor analysis. Can J Psychiatry.

[19] Bland JM, Altman DG (1997). Cronbach's alpha. BMJ.

[20] Tzelepis F, Clinton-McHarg T, Paul CL, Sanson-Fisher RW, Joshua D, Carey ML (2018). Quality of patient-centered care provided to patients attending hematological cancer treatment centers. Int J Environ Res Public Health.

[21] Ferreira DC, Vieira I, Pedro MI, Caldas P, Varela M (2023). Patient satisfaction with healthcare services and the techniques used for its assessment: a systematic literature review and a bibliometric analysis. Healthcare (Basel).

[22] Karaferis DC, Niakas DA (2024). Exploring inpatients’ perspective: a cross-sectional survey on satisfaction and experiences in Greek hospitals. Healthcare (Basel).

